# Description of the First Four Species of the Genus *Pseudogymnoascus* From Antarctica

**DOI:** 10.3389/fmicb.2021.713189

**Published:** 2021-11-19

**Authors:** Pablo Villanueva, Ghislaine Vásquez, Carlos Gil-Durán, Vicente Oliva, Anaí Díaz, Marlene Henríquez, Eduardo Álvarez, Federico Laich, Renato Chávez, Inmaculada Vaca

**Affiliations:** ^1^Department of Chemistry, Faculty of Sciences, University of Chile, Santiago, Chile; ^2^Institute of Biomedical Sciences (ICBM), Mycology Unit, Faculty of Medicine, University of Chile, Santiago, Chile; ^3^Departamento de Protección Vegetal, Instituto Canario de Investigaciones Agrarias, Santa Cruz de Tenerife, Islas Canarias, Spain; ^4^Departamento de Biología, Facultad de Química y Biología, Universidad de Santiago de Chile (USACH), Santiago, Chile

**Keywords:** *Pseudogymnoascus*, new species, Antarctica, taxonomy, multi-locus phylogenetic analyses

## Abstract

The genus *Pseudogymnoascus* represents a diverse group of fungi widely distributed in different cold regions on Earth. Our current knowledge of the species of *Pseudogymnoascus* is still very limited. Currently, there are only 15 accepted species of *Pseudogymnoascus* that have been isolated from different environments in the Northern Hemisphere. In contrast, species of *Pseudogymnoascus* from the Southern Hemisphere have not yet been described. In this work, we characterized four fungal strains obtained from Antarctic marine sponges. Based on multilocus phylogenetic analyses and morphological characterizations we determined that these strains are new species, for which the names *Pseudogymnoascus antarcticus* sp. nov., *Pseudogymnoascus australis* sp. nov., *Pseudogymnoascus griseus* sp. nov., and *Pseudogymnoascus lanuginosus* sp. nov. are proposed. Phylogenetic analyses indicate that the new species form distinct lineages separated from other species of *Pseudogymnoascus* with strong support. The new species do not form sexual structures and differ from the currently known species mainly in the shape and size of their conidia, the presence of chains of arthroconidia, and the appearance of their colonies. This is the first report of new species of *Pseudogymnoascus* not only from Antarctica but also from the Southern Hemisphere.

## Introduction

The fungal genus *Pseudogymnoascus* (*Pseudeurotiaceae, Thelebolales, Leotiomycetes*) was erected by Raillo (1929) to accommodate *P. roseus* and *P. vinaceus*, two species producing ascomata that Raillo distinguished from *Gymnoascus* based on differences in peridial hyphae. Unfortunately, Raillo did not formally specify a type strain for the genus. Many years later, Samson (1972) synonymized *P. vinaceus* with *P. roseus* and formally designated *P. roseus* CBS 395.65, a strain isolated from an alluvial swamp soil in Great Britain, as the neotype for the genus *Pseudogymnoascus*, which has been accepted to date.

In addition to *P. roseus*, only three additional species of *Pseudogymnoascus* were accepted during the 20th century: *P. caucasicus*, *P. bhattii*, and *P. alpinus* (Cejp and Milko, 1966; Samson, 1972; Müller and von Arx, 1982). Using a polyphasic approach including molecular phylogeny analysis based on ITS and morphological characterizations, Rice and Currah (2006) described two other new species, *P. appendiculatus* and *P. verrucosus*.

The interest in *Pseudogymnoascus* experienced a boost in 2009 when the causal agent of white-nose syndrome (WNS), a lethal disease that affects bats in North America, was discovered to be a novel species of psychrophilic fungus that was identified at that time as *Geomyces destructans* (Blehert et al., 2009; Gargas et al., 2009). The discovery of the fungal origin of WNS led to intense phylogenetic studies of fungi that live in bat caves. As a result, the phylogeny of *Geomyces*, *Pseudogymnoascus*, and other close genera was reorganized, including the transference of three species from other genera to the genus *Pseudogymnoascus*: *P. destructans* (formerly *G. destructans*), *P. pannorum* (formerly *G. pannorum*), and *P. carnis* (formerly *Sporotrichum carnis*) (Minnis and Lindner, 2013). These phylogenetic studies also suggested that caves and similar environments in North America harbor several undescribed species of *Pseudogymnoascus* (Lorch et al., 2013; Minnis and Lindner, 2013). Indeed, three new recently described species named *P. lindneri*, *P. turneri*, and *P. palmeri* were isolated from sediment samples obtained in a mine, a railroad tunnel, and a cave, respectively (Crous et al., 2019; Crous et al., 2020). Recently, in 2020, three additional species of *Pseudogymnoascus*, *P. shaanxiensis*, *P. guizhouensis*, and *P. sinensis*, were described. These species were isolated from soils collected in different locations in China (Zhang et al., 2020). To our knowledge, these are the last species of the genus described to date, thus totaling only 15 accepted species of *Pseudogymnoascus* since 1929.

The species of the genus *Pseudogymnoascus* share as common characteristics the ability to produce aleurioconidia and/or arthroconidia and an optimal temperature of growth of approximately 15°C. Concerning sexual structures, several *Pseudogymnoascus* species produce ascomata (Rice and Currah, 2006; Crous et al., 2019; Crous et al., 2020). However, other members of the genus, particularly those transferred from *Geomyces* and *Sporotrichum*, do not produce ascomata (Minnis and Lindner, 2013; Zhang et al., 2020).

Although genus *Pseudogymnoascus* had a broad geographic distribution in cold regions around the world (Gomes et al., 2018; Robicheau et al., 2019; Gupta et al., 2020; Rafiq et al., 2020), all the currently accepted species of *Pseudogymnoascus* were isolated from samples collected in the Northern Hemisphere, mainly in North America. In contrast, new species of *Pseudogymnoascus* obtained in the Southern Hemisphere have not yet been described.

Antarctica is one of the most remote and cold regions on Earth. In this continent, *Pseudogymnoascus* is prevalent. Numerous strains of this fungal genus have been isolated from different Antarctic environments, including terrestrial soils (Godinho et al., 2015; Gomes et al., 2018; Durán et al., 2019; Santos et al., 2020), plants (Santiago et al., 2012; Costa Coelho et al., 2021), shallow-water and deep-sea sediments (Vieira et al., 2018; Wentzel et al., 2019; Ogaki et al., 2020a; Ogaki et al., 2021), macroalgae (Godinho et al., 2013; Furbino et al., 2014; Furbino et al., 2018), sponges (Henríquez et al., 2014), and lakes (Gonçalves et al., 2012; Ogaki et al., 2020b). These results suggest that the Antarctic environments could constitute important reservoirs for new species of *Pseudogymnoascus* not yet described.

Our research team is interested in the study of filamentous fungi from Antarctica. In recent years, we have gathered an important collection of fungal isolates of Antarctic origin, and according to preliminary data, several of these isolates could be new species (Henríquez et al., 2014). Thus, in this work, we described four new species of *Pseudogymnoascus* obtained from samples of Antarctic marine sponges, for which the names *Pseudogymnoascus antarcticus*, *Pseudogymnoascus australis*, *Pseudogymnoascus griseus*, and *Pseudogymnoascus lanuginosus* are proposed. These species represent not only the first four *Pseudogymnoascus* species from Antarctica but also the first four species of this fungal genus from the Southern Hemisphere.

## Materials and Methods

### Fungal Isolates

The fungal isolates analyzed in this work were obtained from sponge samples collected in Fildes Bay, King George Island, South Shetland Islands, Antarctica (Henríquez et al., 2014). Sponge samples were collected at a depth of 6 m. *P. antarcticus* was isolated from a sponge from the genus *Tedania*, *P. lanuginosus* was isolated from a sponge from the order Poecilosclerida, and *P. australis* and *P. griseus* were isolated from two different sponges from the genus *Hymeniacidon* (Henríquez et al., 2014).

The sampling was authorized by the Chilean Antarctic Institute (authorization no. FORE-LO T_15-09, issued on October 13, 2009) and carried out during the XLVI Antarctic Scientific Expedition (ECA46) in December 2009. The holotypes were deposited in the Chilean Fungal Collection (CHFC-EA) of the University of Chile (Santiago, Chile).

### Morphological Analyses

The characteristics of colonies, including the presence of soluble pigments and/or exudates, the obverse and reverse colors of colonies, and the color of mycelia, were recorded after 28 days of growth on oatmeal agar (OA; Difco Laboratories), corn meal agar (CMA; Difco Laboratories), and Sabouraud agar (SBA; Difco Laboratories) at five different temperatures (5, 15, 20, 25, and 37°C). In addition, colony diameters were measured every 5 days, and colonial growth rates were determined.

To induce the formation of ascomata, fungi were grown on CMA, OA, SBA, and potato dextrose agar (PDA; Difco Laboratories) at the same five temperatures mentioned above under two different light conditions: constant darkness and daily cycles of 12 h with fluorescent light alternating with 12 h of darkness. The presence of sexual structures was checked at 10 days and at 2, 4, 6, and 8 months of cultivation.

Light microscopy images were obtained using a Leica DM 2000 LED microscope equipped with an MC170 HD camera and Leica Application Suite (LAS) v.4.8.0 software. Microscopic characteristics were studied on CMA slide cultures incubated for 7 and 14 days at 15°C and mounted in Shear’s solution. Electron microscopy images of *Pseudogymnoascus* conidia were acquired with a Hitachi HT7700 transmission electron microscopy (TEM).

### DNA Extraction, Amplification of Markers, and Sequencing

Each strain was grown for 3 days in CM liquid medium (Gil-Durán et al., 2015). After that, the mycelia were collected, and genomic DNA was isolated as previously described (Mahuku, 2004). Genomic DNA was used for the amplification of five markers using primers and PCR conditions that have been previously described (Minnis and Lindner, 2013). The markers were internal transcribed spacer (ITS), nuclear large subunit (LSU) rDNA, DNA replication licensing factor (MCM7), RNA polymerase II second largest subunit (RPB2), and translation elongation factor EF-1a (TEF1). The sequence of primers used to amplify these markers is described in Supplementary Table 1. The PCR products obtained were purified using a Qiaquick^®^ PCR Purification Kit (Qiagen) and were sequenced at Macrogen Inc. (South Korea).

The sequences generated in this work were deposited in the GenBank database and their accession numbers are listed in Supplementary Table 2.

### Phylogenetic Analyses

By combining the sequences generated in this work with sequences downloaded from GenBank, a sequence dataset was generated (Supplementary Table 2). Alignments for each individual locus were performed using MAFFT v.7^1^ with default parameters (Katoh and Standley, 2013). Both sequence edition and concatenation were performed using Mesquite V3.61 (Maddison and Maddison, 2019). Minnis and Lindner (2013) observed that LSU and TEF1 introns have limited phylogenetic value because they are present and scattered among unrelated members of *Pseudogymnoascus.* Therefore, homologous gaps corresponding to LSU and TEF1 introns were excluded. In addition, non-overlapping ends of sequences in each alignment were trimmed. Gene concordance was assessed for all generated matrices using the “hompart” command in PAUP4.0b10 (Swofford, 2002). The final alignment was deposited in TreeBASE.^2^

Phylogenetic analyses were performed using maximum likelihood (ML) and Bayesian inference (BI) methods. ML analyses were performed with IQ-TREE v.1.6.12 (Nguyen et al., 2015). The best-fit nucleotide substitution model for each locus was estimated using IQ-TREE’s ModelFinder function (Kalyaanamoorthy et al., 2017) following the Bayesian information criterion (BIC). Bootstrap analyses were performed using the ultrafast bootstrap approximation (Minh et al., 2013) with 1,000 replicates. BI analyses were performed with MrBayes v.3.2. (Ronquist et al., 2012). The analyses included two independent runs of 2 million generations with four chains each.

The substitution model was set to GTR + I + G, and the first 25% of samples and trees were discarded as burn-in. The remaining trees were used to construct a 50% majority rule consensus tree.

## Results

### Phylogenetic Analyses

After the exclusion of non-overlapping ends and LSU and TEF1 introns (393 and 57 nucleotides, respectively), the concatenated alignment contained 3,152 nucleotides (ITS: 442; LSU: 931; MCM7: 503 bp; RPB2: 526, and TEF1: 750).

**FIGURE 1:**
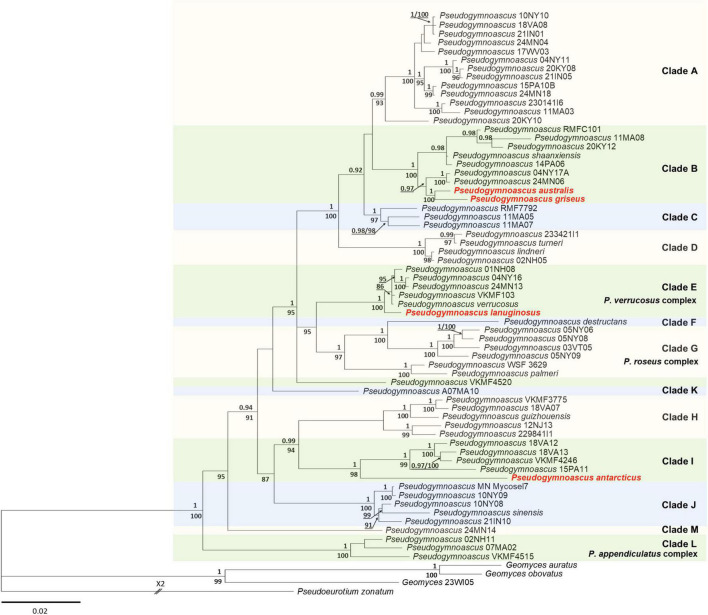
Bayesian inference phylogenetic tree of *Pseudogymnoascus* generated from the concatenated dataset of five loci (ITS, LSU, TEF1, RPB2, and MCM7). Bayesian posterior probabilities (BPPs) are indicated above branches and significant ML bootstrap (BS) values below branches. Only clades that received ≥80% BS and ≥0.95 BPP simultaneously were considered strongly supported and presented at the nodes. Clades are identified using clade nomenclature (A to M) formally defined by Minnis and Lindner (2013). The scale bar indicates 0.02 nucleotide changes per site. The new species are highlighted in bold and red.

The best-fit evolutionary models for each locus in the ML analysis were K2P + R2 (ITS), TNe + R2 (LSU), K2P + I + G4 (MCM7), K2P + G4 (RPB2), and TNe + R3 (TEF1). The tree topology of the BI agreed with that of the ML tree. Therefore, only the BI tree is shown (Figure 1). The phylogenies indicated that all the new species of *Pseudogymnoascus* of Antarctic origin were placed in distinct branches, forming clades separated from other species, with strong bootstrap support (Figure 1). *P. australis*, *P. griseus*, and *P. lanuginosus* were included within the well-supported crown group (clades A–G, PP = 1) (Figure 1). *P. australis* and *P. griseus* clustered together and were placed within clade B, while *P. lanuginosus* was clustered as a sister taxon of clade E (Figure 1). Finally, *P. antarcticus* was clustered as a sister taxon to clade I, forming an independent and well-supported lineage (Figure 1).

### Taxonomy

Phylogenetic analyses based on five gene markers showed that *P. antarcticus*, *P. australis*, *P. griseus*, and *P. lanuginosus* are phylogenetically distinct from the other known species of the genus *Pseudogymnoascus*. Furthermore, the new taxa can be distinguished from each other and all other *Pseudogymnoascus* species based on morphology. These results provide support to describe the four taxa analyzed in this work as new species as follows.

### *Pseudogymnoascus antarcticus* Vaca & R. Chávez, sp. nov.

**FIGURE 2:**
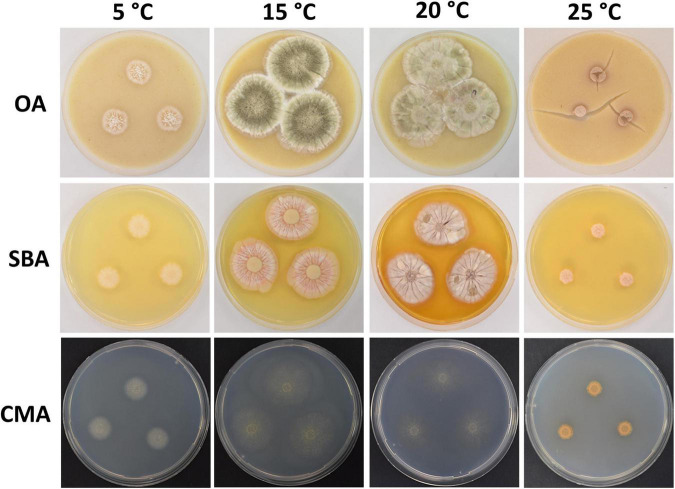
Colony morphology of *Pseudogymnoascus antarcticus* sp. nov. grown on OA, SBA, and CMA media at 5, 15, 20, and 25°C after 28 days of incubation. The cracked agar observed in OA at 25°C was not produced by a technical pitfall, but was produced by the growth of the fungus. Apparently, under these conditions, the fungus extracts most moisture, contracts agar, and cracks it. This was always observed in this medium at this temperature.

**FIGURE 3:**
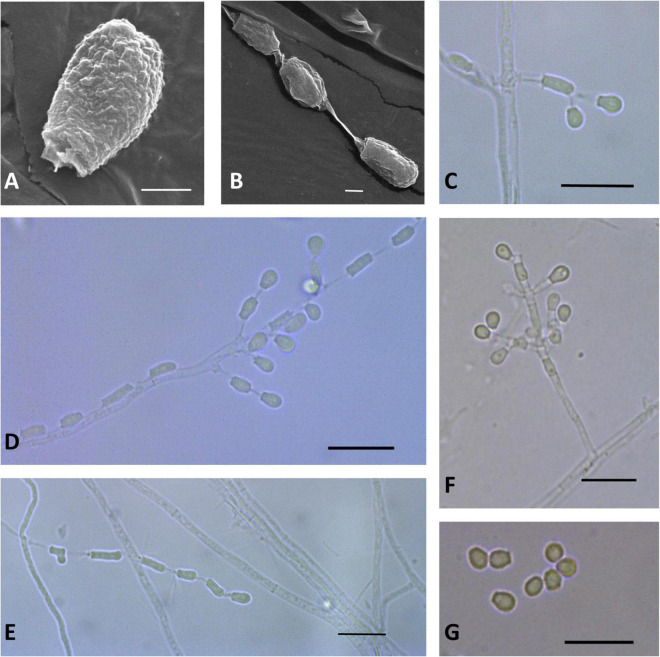
Microscopic analysis of *Pseudogymnoascus antarcticus* sp. nov. Conidium **(A)**; Fertile hyphae bearing arthroconidia and aleurioconidia, sessile, or stalked **(B,C,D,E)**; Conidiophore **(F)**; Conidia **(G)**. In panels **(A,B)**, the structures were observed using transmission electron microscopy, while in panels **(C–G)**, light microscopy was used. Scale bars = 1 μm **(A,B)**, 10 μm **(C–G)**.

MycoBank number: MB 838958 (Figures 2, 3).

Etymology: The name refers to Antarctica, the continent where this species was isolated.

Typus: Antarctica, South Shetland archipelago, King George Island, Fildes Bay, from a marine sponge, 7 Dic. 2009, I. Vaca F09-T2-1 (holotype CHFC-EA 569, stored in a metabolically inactive state in Chilean Fungal Collection).

Description: Hyphae forming bundles with two to six hyphae oriented in parallel, branched, septate, hyaline, smooth, 1.8–2.1 μm wide. Fertile hyphae bearing arthroconidia and/or aleurioconidia, sessile, or stalked. Conidiophores sparse, solitary, erect, hyaline, smooth, narrow, arising laterally from the hyphae, usually bearing verticils of two to four branches, arising from the stipe at an acute angle. Conidia sparse in SBA, more abundant in CMA, intercalary or terminal, off-white *en masse*, hyaline, microtuberculate. Aleurioconidia obovoid to subglobose, occasionally pyriform or clavate, with a broad truncate basal scar, 3.3–5.2 × 2.4–3.4 μm (*av* = 4.0 × 2.8 μm, *n* = 50), in conidiophores separated by connective cells. Intercalary conidia are similar to arthroconidia in shape and size. Arthroconidia intercalary, subglobose to elongate and barrel-shaped, separated by connective cells that undergo rhexolysis, occasionally bearing sessile conidia, 3.6–6.7 × 1.9–2.1 μm (*av* = 5.2 × 1.9 μm, *n* = 50). Ascomata absent.

Culture characteristics: On OA, colonies reach 35 mm in diameter after 28 days at 15°C, round shape, slightly irregular, dense and slightly umbonate, floccose, with radial grooves, olive green to gray, margin filamentous and white, transparent droplets of exudate on the surface of the colony, diffusible pigments absent; reverse brown. On CMA, colonies reach 46 mm in diameter after 28 days at 15°C, appressed, colorless, whitish at the center, consisting of immersed and hyaline hyphae, diffusible pigments and/or exudates absent; reverse colorless. On SBA, colonies reach 35 mm in diameter after 28 days at 15°C, round shape, sometimes irregular, velvety, with radial grooves and smooth center, pale pink with peach color at the center, soluble pigment yellow, exudates absent; reverse orange. Growth occurs in a range of temperatures on OA, CMA, and SBA. Optimum growth was observed at 15 and 20°C, and more reduced growth was observed at 5 and 25°C. At 37°C, small pinpoint colonies are observed.

GenBank accession numbers: ITS = JX845280, LSU = MN417282, MCM7 = MN432493, TEF1-α = MN418131, RPB2 = MN418135.

Notes: In previous phylogenetic analyses using North American strains, Minnis and Lindner (2013) proposed multiple clades of *Pseudogymnoascus* (clades A to M). Figure 1 shows that clade I harbors four strains (15PA11, 18VA12, 18VA13, and VKM F-4246) that remain unidentified species to date (Minnis and Lindner, 2013; Leushkin et al., 2015). *P. antarcticus* represents the most basal branch within clade I, forming an independent lineage with strong support (Figure 1). The closest known species to *P. antarcticus* is *P. guizhouensis*, which is a member of the neighboring clade H (Zhang et al., 2020). The absence of ascomata and the size and shape of the terminal conidia are characteristics shared in both species. However, *P. guizhouensis* and *P. antarcticus* have marked morphological differences. *P. antarcticus* has fertile hyphae that break into chains of arthroconidia, while *P. guizhouensis* lacks arthroconidia. In addition, *P. antarcticus* has subglobose to elongate, barrel-shaped intercalary conidia, while the intercalary conidia of *P. guizhouensis* are cuneiform, barrel-shaped, and markedly smaller than the intercalary conidia of *P. antarcticus*. Finally, conidia of *P. antarcticus* are microtuberculate, while conidia of *P. guizhouensis* are smooth or echinulate.

### *Pseudogymnoascus australis* Vaca & R. Chávez, sp. nov.

**FIGURE 4:**
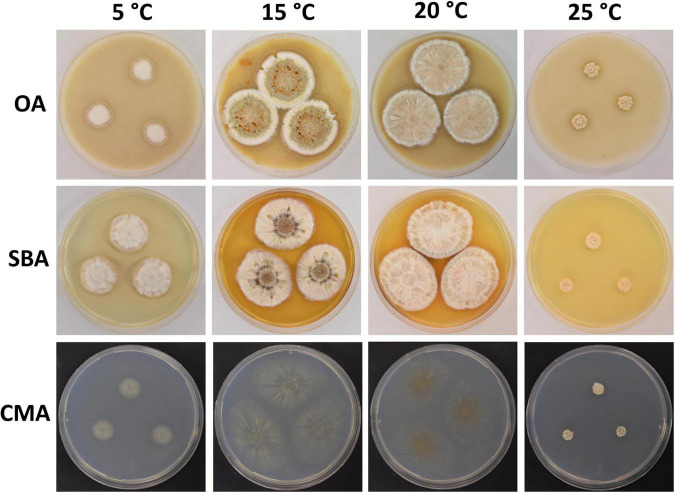
Colony morphology of *Pseudogymnoascus australis* sp. nov. grown on OA, SBA, and CMA media at 5, 15, 20, and 25°C after 28 days of incubation.

**FIGURE 5:**
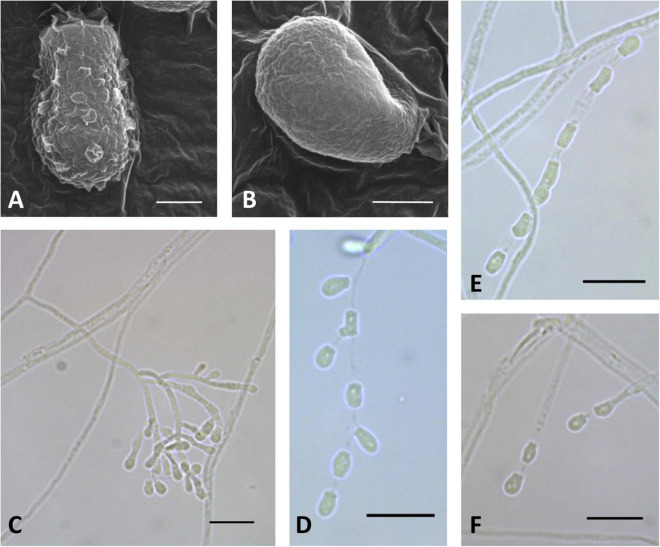
Microscopic analysis of *Pseudogymnoascus australis* sp. nov. Conidia **(A,B)**; Conidiophore **(C)**; Fertile hyphae bearing arthroconidia and aleurioconidia, sessile, or stalked **(D,E,F)**. In panels **(A,B)**, the structures were observed using transmission electron microscopy, while in panels **(C–F)**, light microscopy was used. Scale bars = 1 μm **(A,B)**, 10 μm **(C–F)**.

MycoBank number: MB838968 (Figures 4, 5).

Etymology: The epithet australis means Southern, referring to the region of Earth in which this fungus was isolated.

Typus: Antarctica, South Shetland archipelago, King George Island, Fildes Bay, from a marine sponge, 7 Dic. 2009, I. Vaca F09-T18-3 (holotype CHFC-EA 567, stored in a metabolically inactive state in Chilean Fungal Collection).

Description: Hyphae forming bundles with three to five hyphae oriented in parallel, branched, septate, hyaline, smooth, 1.2–2.0 μm wide. Some lateral hyphae end in chains of barrel-shaped arthroconidia, sometimes bearing aleurioconidia, sessile, or stalked. Conidiophores abundant, solitary, erect, frequently geniculate, or arising in acute angles with the main axis, hyaline, smooth, usually bearing verticils of two to four branches arising from the stipe at an acute angle. Conidia sparse in SBA, more abundant in CMA, intercalary or terminal, off-white *en masse*, hyaline, smooth to rough, irregularly warty. Aleurioconidia obovoid to subglobose, occasionally pyriform or clavate, with a broad truncate basal scar, 3.4–5.6 × 2.3–3.3 μm (*av* = 4.2 × 2.8 μm, *n* = 50), in conidiophores separated by connective cells; intercalary conidia similar to arthroconidia in shape and size. Arthroconidia intercalary subglobose to elongate and barrel-shaped, 3.4–6.8 × 1.3–3.1 μm (*av* = 4.7 × 2.1 μm, *n* = 50), separated by connective cells that undergo rhexolysis, occasionally bearing sessile conidia; arthroconidia formed in hyphal junctions are L- or boot-shaped. Ascomata absent.

Culture characteristics: On OA, colonies reach 48 mm in diameter after 28 days at 15°C, irregular, partially serrated, slightly raised, floccose, grayish, white at the outermost part of the colony, margin white and filiform, abundant exudates in the form of transparent, cinnamon-color droplets of large size, diffusible pigments absent; reverse brown. On CMA, colonies reach 44 mm in diameter after 28 days at 15°C, filamentous, appressed, colorless to whitish, consisting of immersed, hyaline and somewhat shiny hyphae, diffusible pigments and/or exudates absent; reverse colorless. On SBA, colonies reach 44 mm in diameter after 28 days at 15°C, irregular, raised, velvety, with radial grooves, white to beige, filamentous margin, exudates in the form of transparent, cinnamon-color droplets, diffusible pigments not observed; reverse brown. Growth occurs in a range of temperatures on OA, CMA, and SBA. Optimum growth was observed at 15 and 20°C, and more reduced growth was observed at 5 and 25°C. No growth was observed at 37°C.

GenBank accession numbers: ITS = MN417287, LSU = MN417284, MCM7 = MN432491, TEF1-α = MN418133, RPB2 = MN418137.

Notes: *P. australis* was placed as a member of clade B (Figure 1). Clade B is composed of seven taxa: *P. shaaxiensis* (Zhang et al., 2020) and six other strains that remain unidentified species (RMFC101, 14PA06, 20KY12, 04NY17A, 24MN06, and 11MA08) (Minnis and Lindner, 2013). Phylogenetic analysis clearly shows that *P. australis* forms a distinct lineage with strong support (Figure 1). *P. australis* can be differentiated from *P. shaaxiensis* by the size and shape of intercalary conidia (4.7 × 2.1 μm, subglobose to elongate and barrel-shaped vs. 3.5 × 3.0 μm, subglobose, pyriform, or irregularly shaped, respectively). In addition, conidia of *P. australis* may have warts, while conidia of *P. shaaxiensis* are smooth. On the other hand, *P. australis* has fertile hyphae that break into chains of arthroconidia, while *P. shaaxiensis* lacks arthroconidia. Finally, phylogenetic analysis suggested that *P. australis* and the novel species *P. griseus* (see below) are related (Figure 1). However, they can be distinguished based on several morphological characteristics, which are detailed in the protologue of *P. griseus* (see below).

### *Pseudogymnoascus griseus* Vaca & R. Chávez, sp. nov.

**FIGURE 6:**
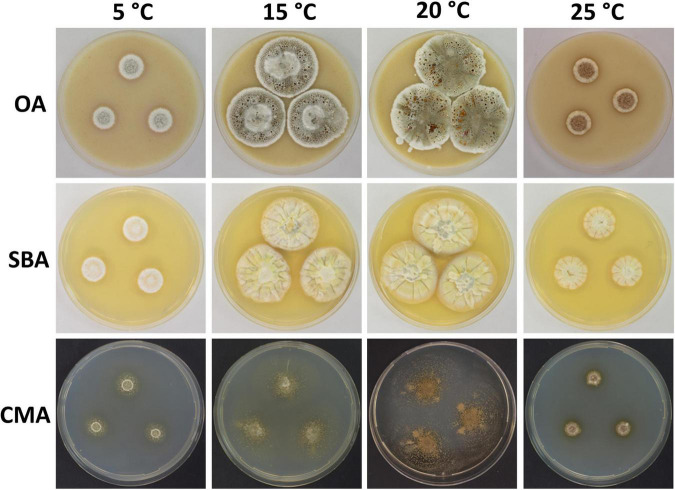
Colony morphology of *Pseudogymnoascus griseus* sp. nov. grown on OA, SBA, and CMA media at 5, 15, 20, and 25°C after 28 days of incubation.

**FIGURE 7:**
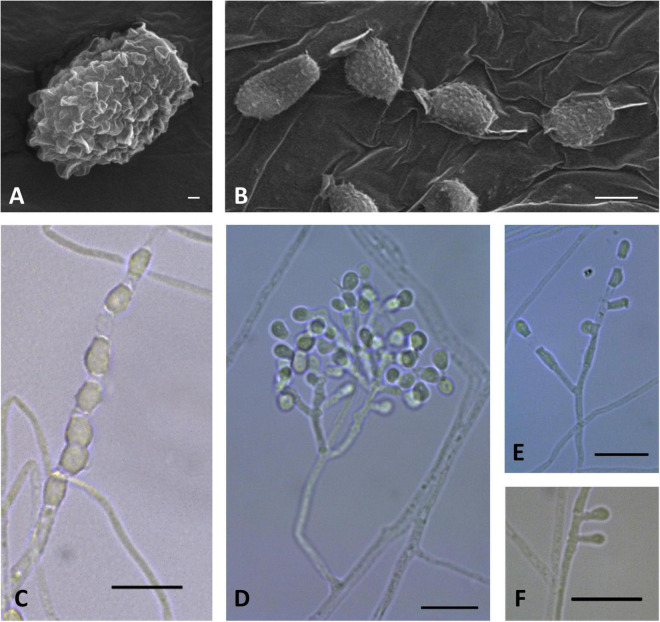
Microscopic analysis of *Pseudogymnoascus griseus* sp. nov. Conidium **(A)**; Chain of arthroconidia **(B,C)**; Conidiophore **(D)**; Fertile hyphae bearing arthroconidia and aleurioconidia sessile or stalked **(E)**; Lonely stalked aleurioconidia **(F)**. In panels **(A,B)**, the structures were observed using transmission electron microscopy, while in panels **(C–F)**, light microscopy was used. Scale bars = 200 nm **(A)**, 2 μm **(B)**, and 10 μm **(C–F)**.

MycoBank number: MB 838969 (Figures 6, 7).

Etymology: The name refers to the gray coloration of the colonies and conidia of this fungus.

Typus: Antarctica, South Shetland archipelago, King George Island, Fildes Bay, from a marine sponge, 7 Dic. 2009, I. Vaca F09-T18-14 (holotype CHFC-EA 568, stored in a metabolically inactive state in Chilean Fungal Collection).

Description: Hyphae forming bundles with two to eight hyphae oriented in parallel, branched, septate, hyaline, smooth, 1.6–2.3 μm wide. Some lateral hyphae end in short chains of arthroconidia, lonely aleurioconidia on fertile hyphae, sessile, or stalked. Conidiophores abundant, erect or geniculated, hyaline, smooth, arising laterally from the hyphae, usually bearing verticils of two to four branches arising from the stipe at an acute angle. Conidia abundant in SBA and CMA, intercalary or terminal, gray to olive green, hyaline, verrucose. Aleurioconidia obovoid to subglobose, occasionally clavate, with broad truncate basal scar, 3.1–4.8 × 2.0–4.0 μm (*av* = 3.8 × 2.8 μm, *n* = 50), in conidiophores separated by connective cells. Intercalary conidia are similar to arthroconidia in shape and size. Arthroconidia intercalary, subglobose to elongate, and barrel-shaped, 3.5–9.6 × 1.7–3.9 μm (*av* = 5.7 × 2.7 μm, *n* = 50), separated by connective cells that undergo rhexolysis, some adhering in pairs. Ascomata absent.

Culture characteristics: On OA, colonies reach 40 mm in diameter after 28 days at 15°C, round, slightly irregular, raised, floccose, gray, white at the center and the outermost part of the colony, margin white and filiform, abundant exudates in the form of transparent droplets of large size, diffusible pigments not observed; reverse gray. On CMA, colonies reach 45 mm in diameter after 28 days at 15°C, filamentous, flat, consisting of immersed and hyaline hyphae, cottony and white at the center, small spots of cottony aerial mycelium emerging in the outermost part of the colony, exudate transparent and scarce, diffusible pigments absent; reverse beige. On SBA, colonies reach 42 mm in diameter after 28 days at 15°C, irregular, raised, floccose, smooth at the center, with radially and cerebriform grooves, beige to yellow and gray, notoriously gray into the grooves, margin irregular and filiform, exudate transparent at the center, diffusible pigments absent; reverse brown. Growth occurs in a range of temperatures on OA, CMA, and SBA. Optimum growth was observed at 15 and 20°C, and more reduced growth was observed at 5 and 25°C. No growth was observed at 37°C.

GenBank accession numbers: ITS = MN417288, LSU = MN417285, MCM7 = MN432492, TEF1-α = MN418134, RPB2 = MN418138.

Notes: *P. griseus* is phylogenetically related to *P. australis*; however, the two species are separated with strong bootstrap support (Figure 1). In addition, they are distinguished by the appearance of the colonies on SBA: colonies of *P. griseus* are floccose, gray, and produce transparent exudates, while colonies of *P. australis* are velvety, white to beige, and produce cinnamon-color exudates. *P. griseus* and *P. australis* also differ in the color, ornamentation, and size of conidia. Conidia from *P. griseus* are gray to olive green and verrucose, while conidia from *P. australis* are smooth to irregularly warty and off-white *en masse*. Aleurioconidia of *P. griseus* are smaller than those of *P. australis* (3.8 × 2.8 μm vs. 4.2 × 2.8 μm); in contrast, arthroconidia of *P. griseus* are larger than those of *P. australis* (5.7 × 2.7 vs. 4.7 × 2.1 μm). With respect to *P. shaaxiensis* (Zhang et al., 2020), *P. griseus* can be differentiated by its gray, verrucose, subglobose to elongate, and barrel-shaped intercalary conidia (the intercalary conidia of *P. shaaxiensis* are colorless, smooth, subglobose, pyriform, or irregularly shaped). Another important difference is that *P. shaaxiensis* lacks chains of arthroconidia, which are abundant in *P. griseus*.

### *Pseudogymnoascus lanuginosus* Vaca & R. Chávez, sp. nov.

**FIGURE 8:**
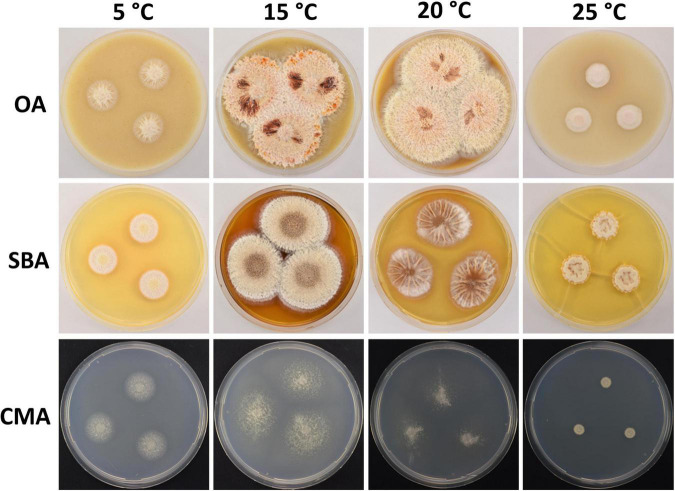
Colony morphology of *Pseudogymnoascus lanuginosus* sp. nov. grown on OA, SBA, and CMA media at 5, 15, 20, and 25°C after 28 days of incubation. The cracked agar observed in SBA at 25°C was not produced by a technical pitfall, but was produced by the growth of the fungus. Apparently, under these conditions, the fungus extracts most moisture, contracts agar, and cracks it. This was always observed in this medium at this temperature.

**FIGURE 9:**
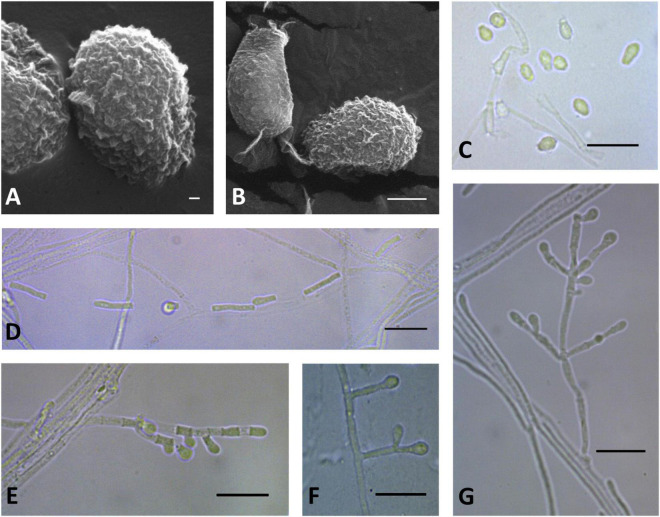
Microscopic analysis of *Pseudogymnoascus lanuginosus* sp. nov. Conidia **(A,B,C)**; Chain of arthroconidia **(D)**; Fertile hyphae bearing arthroconidia and aleurioconidia, sessile, or stalked **(E)**; Stalked aleurioconidia **(F)**; Conidiophore **(G)**. In panels **(A,B)**, the structures were observed using transmission electron microscopy, while in panels **(C–G)**, light microscopy was used. Scale bars = 200 nm **(A)**, 1 μm **(B)**, and 10 μm **(C–G)**.

MycoBank number: MB 838970 (Figures 8, 9).

Etymology: The name refers to the woolly appearance of the colonies.

Typus: Antarctica, South Shetland archipelago, King George Island, Fildes Bay, from a marine sponge, 7 Dic. 2009, I. Vaca F09-T18-27 (holotype CHFC-EA 570, stored in a metabolically inactive state in Chilean Fungal Collection).

Description: Hyphae forming abundant bundles with three to eight hyphae oriented in parallel, branched, septate, hyaline, smooth, 0.9–1.4 μm wide. Spiral hyphae are usually found. Fertile hyphae bearing arthroconidia and/or aleurioconidia, sessile, or stalked. Conidiophores sparse, solitary, sometimes minimally differentiated from hyphae, hyaline, smooth, arising from the hyphae erect or geniculated, usually bearing verticils of two to four branches at an acute angle. Conidia absent in SBA, abundant in CMA, intercalary or terminal, variable in size and shape, off-white *en masse*, hyaline, tuberculate, sparsely ornamented with minute warts; preponderance of intercalary arthroconidia. Aleurioconidia clavate, occasionally obovoid to subglobose, with the apex rounded and broad truncate basal scar, 2.2–5.3 × 2.1–3.1 μm (*av* = 4.3 × 2.6 μm, *n* = 50). Intercalary conidia are similar to arthroconidia in shape and size. Arthroconidia intercalary, barrel-shaped or cylindrical, 4.9 –14.5 × 1.4–2.3 μm (*av* = 8.6 × 1.7 μm, *n* = 50), separated by connective cells that undergo rhexolysis; occasionally bearing sessile conidia; arthroconidia formed in hyphae junctions are L- or boot-shaped. Ascomata absent.

Culture characteristics: On OA, colonies reach 45 mm in diameter after 28 days at 15°C, irregular, raised, aerial mycelium abundant, appearing as woolly tufts, pale pink to brown, margin white and filamentous, abundant exudates in the form of droplets of cinnamon or brick-red color, diffusible pigments absent; reverse brown. On CMA, colonies reach 45 mm in diameter after 28 days at 15°C, round, flat, cottony, white, consisting of immersed and hyaline hyphae, exudates and diffusible pigments absent; reverse white. On SBA, colonies reach 45 mm in diameter after 28 days at 15°C, round, raised, aerial mycelium abundant, appearing as woolly tufts, surface with concentric circles brown, beige, and white (from center to edge), margin entire and filamentous, exudates absent, soluble pigment cinnamon; reverse brown in center, white in outer region. Growth occurs in a range of temperatures on OA, CMA, and SBA. Optimum growth was observed at 15 and 20°C, and more reduced growth was observed at 5 and 25°C. No growth was observed at 37°C.

GenBank accession numbers: ITS = MN417286, LSU = MN417283, MCM7 = MN418139, TEF1-α = MN418132, RPB2 = MN418136.

Notes: *P. lanuginosus* was placed as the most basal member of clade E (Figure 1). Clade E is composed of four strains that remain unidentified species (01NH08, 04NY16, 24MN13, and VKM F-103) (Minnis and Lindner, 2013; Leushkin et al., 2015) and *P. verrucosus* (Rice and Currah, 2006). Phylogenetic analysis clearly shows that *P. lanuginosus* forms a distinct lineage with strong support (Figure 1). *P. lanuginosus* can be differentiated from *P. verrucosus* by the absence of ascomata, the woolly appearance and the pink coloration of its colonies on OA, and its more elongated arthroconidia (8.6 × 1.7 μm vs. 2.5–5 × 2–3 μm).

## Discussion

*Pseudogymnoascus* is a fungal genus frequently found in terrestrial and marine samples collected in Antarctica (Santiago et al., 2015; Gomes et al., 2018; Rosa et al., 2019; Carvalho et al., 2020; Ogaki et al., 2020a, b; Rosa et al., 2020). Most of the *Pseudogymnoascus* isolates obtained from these samples have not been identified to the species level. To date, novel species of *Pseudogymnoascus* of Antarctic origin have not been described. Hence, the four new species *P. antarcticus*, *P. australis*, *P. griseus*, and *P. lanuginosus* described in this work represent the first four species of *Pseudogymnoascus* from Antarctica and the Southern Hemisphere.

The new species were characterized through phylogenetic and morphological analyses. The phylogenetic analyses support the placement of *P. australis* and *P. griseus* as members of clade B of *Pseudogymnoascus*, which also includes *P. shaaxiensis*, the only species thus far described within this clade (Figure 1). The three species share the absence of ascomata as a common characteristic, suggesting, in principle, that clade B could harbor strains of *Pseudogymnoascus* that do not present sexual structures. An analysis of the sexual development of additional strains of this clade is required to confirm this hypothesis. Concerning ecological characteristics, all of the current members of clade B were isolated from soil samples from different origins: bat hibernacula soil in the eastern United States (strains 14PA06, 20KY12, 04NY17A, 24MN06, and 11MA08; Lorch et al., 2013), desert grassland soil in Utah (RMFC101; Minnis and Lindner, 2013) and epiphytic soil in China (*P. shaaxiensis*; Zhang et al., 2020). In contrast, *P. australis* and *P. griseus* were obtained from Antarctic marine sponges. As a member of class *Leotiomycetes*, *Pseudogymnoascus* is expected to have a worldwide distribution in diverse environments, including soil, fresh and marine water, or air (Johnston et al., 2019). Therefore, the placement of *P. australis* and *P. griseus* in clade B suggests that the diversity currently represented in clade B would be only a small fraction of the total diversity of *Pseudogymnoascus* of this clade.

*Pseudogymnoascus lanuginosus* was placed as a member of clade E of *Pseudogymnoascus*. This clade also includes strains 01NH08, 04NY16, 24MN13, and VKM F-103, and the species *P. verrucosus* (Rice and Currah, 2006; Minnis and Lindner, 2013; Leushkin et al., 2015). Unlike *P. lanuginosus*, *P. verrucosus* produces ascomata (Rice and Currah, 2006), indicating that clade E could harbor species of *Pseudogymnoascus* that do or do not produce sexual structures. This is in agreement with previous observations by Minnis and Lindner (2013), who noted that *P. destructans*, a species where the sexual stage has not been observed, was placed among a number of lineages with known sexual stages. Regarding the ecological origin of strains within clade E, *P. verrucosus* was isolated from a brown-rotted black spruce wood found in a *Sphagnum* bog in the southern boreal forest of western Canada (Rice and Currah, 2006), strain VKM F-103 was isolated from soil in New York, United States (Leushkin et al., 2015), and strains 01NH08, 04NY16, and 24MN13 were isolated from bat hibernacula soil in the eastern United States (Lorch et al., 2013). Therefore, clade E harbors a diverse group of *Pseudogymnoascus* that includes wood-degrading fungi, soil fungi, and fungi associated with marine sponges.

Finally, the phylogenetic position of *P. antarcticus* shows that this species represents an independent lineage, which is related to clade I. This clade includes three strains (18VA12, 18VA13, and 15PA11) isolated from bat hibernacula soil in the eastern United States (Lorch et al., 2013), and strain VKM F-4246 isolated from coniferous litter in Selenge Aimag, Mongolia (Leushkin et al., 2015), which remain undescribed species. Thus, the diversity of *Pseudogymnoascus* currently represented in clade I is limited and an analysis of additional strains is required to reveal the total diversity of this clade.

It is interesting to note that none of the new species described in this work present sexual structures. In Ascomycota, sexual reproduction is often difficult to detect under laboratory conditions (Ni et al., 2011). In the case of *Pseudogymnoascus*, some species produce sexual structures (Rice and Currah, 2006; Crous et al., 2019; Crous et al., 2020). However, a significant number of strains of *Pseudogymnoascus*, including some accepted species, have not shown evidence of sexual reproduction (Chatuverdi et al., 2010; Leushkin et al., 2015; Zhang et al., 2020).

Asexual structures of the new species usually consist of conidiophores bearing intercalary and terminal conidia, lonely aleurioconidia, and chains of arthroconidia. Conidiophores bearing intercalary and terminal conidia are a general characteristic observed in several species of *Pseudogymnoascus*. In contrast, chains of arthroconidia have been previously observed only in *P. appendiculatus* (Rice and Currah, 2006) and *P. destructans* (Gargas et al., 2009). Most *Pseudogymnoascus* species have hyaline or white to off-white conidia *en masse*. Thus, the gray to olive green conidia observed in the new species *P. griseus* are a singular characteristic that has not previously been reported in other species of the genus. *P. lanuginosus* can be easily distinguished by its larger arthroconidia. Finally, *P. antarcticus* grows at 37°C, a physiological characteristic that has only been reported before for *P. caucasicus* (Cejp and Milko, 1966).

More research is necessary to achieve a full picture of the taxonomy of *Pseudogymnoascus* of Antarctic origin. In this context, our findings represent a first step, opening the opportunity for the description of additional new species of *Pseudogymnoascus* by other researchers working on fungi from Antarctica.

## Author’s Note

While this manuscript was in review, Zhang et al. (2021) reported other four new species of *Pseudogymnoascus*, isolated from soil in China (*P. catenatus*, *P. fujianensis*, *P. yunnanensis*, and *P. zhejiangensis*). Our species are different to those described in the paper of Zhang et al. (2021).

## Data Availability Statement

The datasets presented in this study can be found in online repositories. The names of the repository/repositories and accession number(s) can be found in the article/Supplementary Material.

## Author Contributions

IV and RC were responsible for conceptualization and drafted the manuscript. IV, FL, EÁ, and GV designed the experiments. PV, GV, VO, AD, and CG-D performed all laboratory analysis and evaluated the data. PV was responsible for data curation and software. All authors contributed to the article and approved the submitted version.

## Conflict of Interest

The authors declare that the research was conducted in the absence of any commercial or financial relationships that could be construed as a potential conflict of interest.

## Publisher’s Note

All claims expressed in this article are solely those of the authors and do not necessarily represent those of their affiliated organizations, or those of the publisher, the editors and the reviewers. Any product that may be evaluated in this article, or claim that may be made by its manufacturer, is not guaranteed or endorsed by the publisher.

## Footnotes

1
http://mafft.cbrc.jp/alignment/server/

2
http://purl.org/phylo/treebase/phylows/study/TB2:S28862
