# Supplementary material for: Description of the First Four Species of the Genus Pseudogymnoascus From Antarctica
Source: Front Microbiol. 2021 Nov 19;12:713189. doi: 10.3389/fmicb.2021.713189 (PMC8640180; doi:10.3389/fmicb.2021.713189)
Supplement: Supplementary file 1 [file Data_Sheet_1.docx]

Supplementary Material

# Supplementary Figures and Tables

**Supplementary Table S1.** Sequences of primers used for the amplification of molecular markers in this study.

| **Molecular marker^a^** | **Primer name** | **Primer sequence (5´-3´)** | **Reference^b^** |
| --- | --- | --- | --- |
| **ITS** | ITS1 | TCCGTAGGTGAACCTGCG | White et al., 1990 |
|  | ITS4 | TCCTCCGCTTATTGATATGC | White et al., 1990 |
| **LSU** | LROR | ACCCGCTGAACTTAAGC | Moncalvo et al., 2000 |
|  | LR7 | TACTACCACCAAGATCT | Vilgalys and Hester, 1990 |
| **TEF1** | 2218R | ATGACACCRACRGCRACRGTYTG | Rehner and Buckley, 2005 |
|  | 983F | GCYCCYGGHCAYCGTGAYTTYAT | Rehner and Buckley, 2005 |
| **MCM7** | Mcm7-709for | ACNMGNGTNTCVGAYGTHAARCC | Schmitt et al., 2009 |
|  | Mcm7-1348rev | GAYTTDGCNACNCCNGGRTCWCCCAT | Schmitt et al., 2009 |
| **RPB2** | fRPB2-7cF | ATGGGYAARCAAGCYATGGG | Liu et al., 1999 |
|  | RPB2-3053bR | TGRATYTTRTCRTCSACCAT | Reeb et al., 2004 |

^a^ ITS: internal transcribed spacer; LSU: nuclear large subunit rDNA; MCM7: DNA replication licensing; RPB2: RNA polymerase II second largest subunit; TEF1: translation elongation factor EF-1a.

^b^ Complete references are: 1) White, T.J., Bruns, T., Lee, S., Taylor, J . (1990). “Amplification and direct sequencing of fungal ribosomal RNA genes for phylogenetics”, in PCR protocols: a guide to methods and application, eds. M.A. Innis, D.H. Gelfand, J.J. Sninsky and T.J. White (Academic Press Inc, San Diego), 315–322. 2) Moncalvo, J.M., Lutzoni, F.M., Rehner, S.A., Johnson, J., Vilgalys, R. (2000). Phylogenetic relationships of agaric fungi based on nuclear large subunit ribosomal DNA sequences. Syst. Biol. 49, 278-305. 3) Vilgalys, R. and Hester, M. (1990). Rapid genetic identification and mapping of enzymatically amplified ribosomal DNA from several Cryptococcus species. J. Bacteriol. 172, 4238-4246. 4) Rehner, S.A. and Buckley, E. (2005). A Beauveria phylogeny inferred from nuclear ITS and EF1-a sequences: evidence for cryptic diversification and links to Cordyceps teleomorphs. Mycologia. 97, 84-98. 5) Schmitt, I., Crespo, A., Divakar, P.K., Fankhauser, J.D., Herman- Sackett E., Kalb, K. et al. (2009). New primers for promising single-copy genes in fungal phylogenetics and systematics. Persoonia. 23, 35-40. 6) Liu, Y.J., Whelen, S., Hall, B.D. (1999). Phylogenetic relationships among ascomycetes: evidence from an RNA polymerase II subunit. Mol. Biol. Evol. 16, 1799-1808. 7) Reeb, V., Lutzoni, F., Roux, C. (2004). Contribution of RPB2 to multilocus phylogenetic studies of the euascomycetes (Pezizomycotina, Fungi) with special emphasis on the lichen-forming Acarosporaceae and evolution of polyspory. Mol. Phylogenet. Evol. 32, 1036-1060.

**Supplementary Table S2.** GenBank accession numbers of the sequences used in this study. Sequences highlighted in bold were generated in this study.

| **Species name** | **Strain^a^** | **GenBank accession numbers^b^** | | | | |
| --- | --- | --- | --- | --- | --- | --- |
|  |  | **ITS** | **LSU** | **MCM7** | **RPB2** | **TEF1** |
| ***Geomyces auratus*** | CBS 108.14 | KF039895 | KF017864 | KF017690 | KF017746 | KF017805 |
| ***Geomyces obovatus*** | CGMCC 3.18491 | MT509362 | MT509376 | MT534202 | MT534216 | MT534227 |
| ***Geomyces* sp.** | 23WI05 | JX270595 | KF017857 | KF017683 | KF017740 | KF017798 |
| ***Pseudeurotium zonatum*** | CBS 329.36  AFTOL-ID 1912 | AY129286 | DQ470988 | na^c^ | DQ470940 | DQ471112 |
| ***Pseudogymnoascus* sp.** | 02NH11 | JX270356 | KF017819 | KF017650 | KF017704 | KF017759 |
| ***Pseudogymnoascus* sp.** | 07MA02 | JX270402 | KF017827 | KF017658 | KF017712 | KF017767 |
| ***Pseudogymnoascus destructans*** | 20631-21 | EU884921 | KF017865 | KF017691 | KF017747 | KF017806 |
| ***Pseudogymnoascus guizhouensis*** | GZUIFR 376.1 | MT509369 | MT509383 | MT534209 | MT534223 | MT534234 |
| ***Pseudogymnoascus* sp.** | 02NH05 | JX270350 | KF017818 | KF017649 | KF017703 | KF017758 |
| ***Pseudogymnoascus* sp.** | 05NY06 | JX270385 | KF017824 | KF017655 | KF017709 | KF017764 |
| ***Pseudogymnoascus* sp.** | 05NY08 | JX270387 | KF017825 | KF017656 | KF017710 | KF017765 |
| ***Pseudogymnoascus* sp.** | 05NY09 | JX270388 | KF017826 | KF017657 | KF017711 | KF017766 |
| ***Pseudogymnoascus roseus*** | WSF 3629 | KF039897 | KF017870 | KF017696 | KF017751 | KF017811 |
| ***Pseudogymnoascus* sp.** | 14PA06 | JX270469 | KF017839 | KF017668 | KF017723 | KF017779 |
| ***Pseudogymnoascus* sp.** | RMF C 101 | KF039896 | KF017869 | KF017695 | KF017750 | KF017810 |
| ***Pseudogymnoascus shaanxiensis*** | GZUIFR HZ5.7 | MT509366 | MT509380 | MT534206 | MT534220 | MT534231 |
| ***Pseudogymnoascus sinensis*** | CGMCC 3.18493 | MT509364 | MT509378 | MT534204 | MT534218 | MT534229 |
| ***Pseudogymnoascus* sp.** | 01NH08 | JX270343 | KF017816 | KF017647 | KF017701 | KF017756 |
| ***Pseudogymnoascus* sp*.*** | 04NY11 | JX270375 | KF017821 | KF017652 | KF017706 | KF017761 |
| ***Pseudogymnoascus* sp*.*** | 04NY16 | JX270377 | KF017822 | KF017653 | KF017707 | KF017762 |
| ***Pseudogymnoascus* sp.** | 04NY17A | JX270378 | KF017823 | KF017654 | KF017708 | KF017763 |
| ***Pseudogymnoascus* sp.** | 10NY08 | JX270432 | KF017829 | KF017659 | KF017714 | KF017769 |
| ***Pseudogymnoascus* sp.** | 10NY09 | JX270433 | KF017830 | KF017660 | KF017715 | KF017770 |
| ***Pseudogymnoascus* sp.** | 10NY10 | JX270434 | KF017831 | na | KF017716 | KF017771 |
| ***Pseudogymnoascus* sp.** | 11MA03 | JX270438 | KF017832 | KF017661 | KF017717 | KF017772 |
| ***Pseudogymnoascus* sp.** | 11MA05 | JX270440 | KF017833 | KF017662 | KF017718 | KF017773 |
| ***Pseudogymnoascus* sp*.*** | 11MA07 | JX270442 | KF017834 | KF017663 | KF017719 | KF017774 |
| ***Pseudogymnoascus* sp.** | 11MA08 | JX270443 | KF017835 | KF017664 | KF017720 | KF017775 |
| ***Pseudogymnoascus* sp.** | 12NJ13 | JX270459 | KF017838 | KF017667 | KF017722 | KF017778 |
| ***Pseudogymnoascus* sp.** | 15PA11 | JX270486 | KF017843 | KF017671 | KF017727 | KF017783 |
| ***Pseudogymnoascus* sp*.*** | 17WV03 | JX270510 | KF017844 | KF017672 | KF017728 | KF017784 |
| ***Pseudogymnoascus* sp.** | 18VA07 | JX270527 | KF017847 | KF017675 | na | KF017788 |
| ***Pseudogymnoascus* sp.** | 18VA08 | JX270528 | KF017848 | KF017676 | KF017731 | KF017789 |
| ***Pseudogymnoascus* sp.** | 18VA12 | JX270532 | KF017849 | na | KF017732 | KF017790 |
| ***Pseudogymnoascus* sp.** | 18VA13 | JX270533 | KF017850 | na | KF017733 | KF017791 |
| ***Pseudogymnoascus* sp.** | 20KY08 | JX270562 | KF017851 | KF017677 | KF017734 | KF017792 |
| ***Pseudogymnoascus* sp.** | 20KY10 | JX270563 | KF017852 | KF017678 | KF017735 | KF017793 |
| ***Pseudogymnoascus* sp.** | 20KY12 | JX270565 | KF017853 | KF017679 | KF017736 | KF017794 |
| ***Pseudogymnoascus* sp.** | 21IN01 | JX270568 | KF017854 | KF017680 | KF017737 | KF017795 |
| ***Pseudogymnoascus* sp.** | 21IN05 | JX270572 | KF017855 | KF017681 | KF017738 | KF017796 |
| ***Pseudogymnoascus* sp.** | 21IN10 | JX270577 | KF017856 | KF017682 | KF017739 | KF017797 |
| ***Pseudogymnoascus* sp.** | 24MN04 | JX270612 | KF017859 | KF017685 | KF017741 | KF017800 |
| ***Pseudogymnoascus* sp.** | 24MN06 | JX270614 | KF017860 | KF017686 | KF017742 | KF017801 |
| ***Pseudogymnoascus* sp.** | 24MN13 | JX270621 | KF017861 | KF017687 | KF017743 | KF017802 |
| ***Pseudogymnoascus* sp.** | 24MN14 | JX270622 | KF017862 | KF017688 | KF017744 | KF017803 |
| ***Pseudogymnoascus* sp*.*** | 24MN18 | JX270626 | KF017863 | KF017689 | KF017745 | KF017804 |
| ***Pseudogymnoascus* sp.** | 22984-1-I1 | JX415262 | KF017866 | KF017692 | na | KF017807 |
| ***Pseudogymnoascus* sp.** | 23014-1-I6 | JX512256 | KF017867 | KF017693 | KF017748 | KF017808 |
| ***Pseudogymnoascus* sp.** | 03VT05 | KF039892 | KF017820 | KF017651 | KF017705 | KF017760 |
| ***Pseudogymnoascus* sp.** | A07MA10 | KF039893 | KF017828 | na | KF017713 | KF017768 |
| ***Pseudogymnoascus* sp.** | 15PA10B | KF039894 | KF017842 | KF017670 | KF017726 | KF017782 |
| ***Pseudogymnoascus* sp.** | RMF 7792 | KF039898 | KF017871 | KF017697 | KF017752 | KF017812 |
| ***Pseudogymnoascus* sp.** | MN-Mycosel-7 | KF039899 | KF017872 | KF017698 | KF017753 | KF017813 |
| ***Pseudogymnoascus* sp.** | VKM F-4246 | JPJU01003588 ^d^ (1 to 491) | JPJU01003588 (560 to 1757) | JPJU01001151  (12991 to 13609) | JPJU01001166  (6540 to 7304) | JPJU0100175  (36501 to 36948) |
| ***Pseudogymnoascus* sp.** | VKM F-103 | JPKB01001524  (1 to 491) | JPKB01001524  (560 to 1413) | JPKB01001753  (13559 to 14177) | JPKB01001744  (6669 to 7432) | JPKB01001786  (12082-12985) |
| ***Pseudogymnoascus* sp.** | VKM F-3775 | JPJT01006801  (2157 to 2734) | JPJT01006801  (2803 to 4000) | JPJT01006817  (1917 to 2535) | JPJT01004817  (6048 to 6811) | JPJT01000524  (8398 to 9301) |
| ***Pseudogymnoascus* sp.** | VKM F-4520 | na | JPKE01003101  (1 to 372) | JPKE01002801  (11023 to 11641) | JPKE01003069  (15190 to 15954) | JPKE01002324  (21914 to 22818) |
| ***Pseudogymnoascus* sp.** | VKM F-4514 | JPJX01002711  (178 to 755) | JPJX01002812  (1 to 973) | JPJX01000952  (6565 to 7183) | JPJX01000486  (13807 to 14571) | JPJX01001337  (38563 to 39010) |
| ***Pseudogymnoascus verrucosus*** | UAMH 10579 | NR_111197 | na | XM_018270150 | XM_018270703 | XM_018277618 |
| ***Pseudogymnoascus griseus*** | CHFC-EA 568 | **MN417288** | **MN417285** | **MN432492** | **MN418138** | **MN418134** |
| ***Pseudogymnoascus lanuginosus*** | CHFC-EA 570 | **MN417286** | **MN417283** | **MN418139** | **MN418136** | **MN418132** |
| ***Pseudogymnoascus australis*** | CHFC-EA 567 | **MN417287** | **MN417284** | **MN432491** | **MN418137** | **MN418133** |
| ***Pseudogymnoascus antarcticus*** | CHFC-EA 569 | **JX845280** | **MN417282** | **MN432493** | **MN418135** | **MN418131** |
| ***Pseudogymnoascus turneri*** | LHU121 | MN542213 | na | na | MN541380 | MN541379 |
| ***Pseudogymnoascus palmeri*** | LHU407 | MT988150 | na | na | MW054468 | MW054467 |
| ***Pseudogymnoascus lindneri*** | LHU158 | MN542212 | na | na | MN541384 | MN541383 |
| ***Pseudogymnoascus verrucosus*** | UAMH 10579 | NR_111197 | na | XM_018270150 | XM_018270703 | XM_018277618 |

^a^ CBS: Culture collection of the Westerdijk Biodiversity Institute, Ultrech, the Netherlands; CGMCC: China General Microbiological Culture Collection Center, Beijing, China; GZUIFR: Herbarium of Guizhou Agricultural College, Institute of Fungus Resources, Guizhou University, China; CHFC: Chilean Fungal Collection, Facultad de Medicina, Universidad de Chile, Santiago, Chile; RMF : Rocky Mountain Fungi, IBT (Institut for Bioteknologi) Culture collection of Fungi, Lyngby, Denmark; WSF: Wisconsin Soil Fungi, IBT (Institut for Bioteknologi) Culture collection of Fungi, Lyngby, Denmark. UAMH: University of Alberta Microfungus Collection and Herbarium (UAMH) Centre for Global Microfungal Biodiversity, University of Toronto, Canada; VKM: All-Russian Collection of Microorganisms, Skryabin Institute of Biochemistry and Physiology of Microorganisms, Russian Academy of Science, Pushchino, Russia.

^b^ ITS: internal transcribed spacer; LSU: nuclear large subunit rDNA; MCM7: DNA replication licensing; RPB2: RNA polymerase II second largest subunit; TEF1: translation elongation factor EF-1a.

^c^ na = not available

^d^ Some markers were extracted from sequenced genomes. In these cases, the GenBank accession number of the contig is given. Numbers in parentheses indicate the location of the sequence of interest in the contig (from nucleotide XX to XX).
